# The Mansion House and the Hospitals

**Published:** 1907-06-01

**Authors:** 


### THE MANSION HOUSE AND THE HOSPITALS.

### THE BANQUET FOR THE NORTH-EASTERN HOSPITAL FOR CHILDREN.

Some captious critics have declared that the Lord Mayor, during his year of office, ought not to use his influence to the fullest extent to promote the welfare and development of particular charities, to whose interest he has devoted a large measure of his time and energies, before he entered the Mansion House. We hold distinctly that every Lord Mayor, if he is to discharge fully the duties which devolve upon him, must make an infinitely better representative of the City during his residence at the Mansion House if he has previously identified himself with institutions and movements for the benefit of his fellows. In such a case he is not only justified, but bound, to use the influence of his position to help forward the work of such institutions during his year of office. A former Lord Mayor, Sir Marcus Samuel, gave a banquet at the Mansion House on behalf of the London Hospital, which was probably the most instructive and helpful dinner ever held on behalf of a great charity.

Sir William Treloar has championed for many years the cause of crippled and injured children. He is the founder of the annual Christmas banquet at the Guildhall to these little sufferers, and in connection with this work has been the means of doing an immense amount of good. He is endeavouring to raise a sum of £60,000 to place this movement upon a permanent and adequate footing, and we hope he may succeed in securing all the money necessary to accomplish this very desirable object. All honour to Sir William Treloar for his noble work on behalf of helpless children, and for his courage in continuing that work energetically during his mayoralty. There is no man or woman of feeling or character in the land to whom the sufferings of children do not appeal, and everybody should send something this year to the Lord Mayor for his fund.

It is matter for congratulation, though not for surprise, that Sir William Treloar should have given a banquet on behalf of the funds of the North-Eastern Hospital at the Mansion House on the 28th ultimo. Of all the children's hospitals the North-Eastern Hospital for Children in the Hackney Road, Bethnal Green, should appeal with accumulated force to every philanthropist who is desirous of giving his money carefully and intelligently. The speakers at the Mansion House had a very easy task. There is no object which lends itself better to true eloquence than the cause of the children, and the North-Eastern Hospital has made so much progress recently that the speakers found no difficulty in making their appeals effective.

When we inspected the hospital a few days ago we found all the children bright and happy, and the wards free from the incessant crying which sometimes marks inefficient administration of a children's ward. The extension of the hospital, with the addition of balconies, have had a marked effect upon the health of the children. These balconies facilitate arrangements whereby the children live much in the open air, and although the outlook is merely on to a main thoroughfare, or roofs and chimney pots, the fact of being continually in the air gives to the town child robustness and vitality, which are so often absent in a great city like London. Indeed a visitor to the wards might well ask himself, on seeing the children, are these patients really ill at all? Another feature of the hospital is the spotless cleanliness and excellent order, testifying to considerable administrative gifts upon the part of the matron and her staff. These features were so marked in the Nurses' Home that we were led to the conclusion that one spotless bath had never been used. On inquiry, however, the Home Sister was able to satisfy us that this bath had been often used, a fact which testifies to the excellence of her method and organisation. One point came out clearly in regard to the Paris picnic and what nurses are likely to see there. One of the French doctors, when shown the excellent home for nurses at the North-Eastern Hospital, expressed his surprise at such accommodation being provided for attendants on the sick. This is of course merely an indication that at the present time there are practically no trained nurses in French hospitals, and that the old bad system under which anybody was thought good enough to attend the sick is still largely prevalent. Paris therefore, at present, is not a field of instruction for trained nurses, but we are glad to think that the warm friendship which now exists between the French and English peoples is calculated to change all this for the better.

Economically the hospital is well administered. It does a great deal of work in circumstances rendered difficult by the want of an adequate out-patient department, and it has one special feature which we regard as a novelty. Owing to a desire to prevent as far as possible the risk of introducing an infectious disease into the wards, no visitors are permitted to see the patients, except in very severe cases. The average residence of each patient is about twenty-three days. The absence of visitors simplifies the work of the hospital, and has proved in all respects beneficial to the patients, and not unpopular with the parents. In the result we understand that the system of excluding visitors is likely to become much more general in connection with children's hospitals in the future, than it has been in the past. The management takes care, by permitting cots to be endowed for life, or in perpetuity, to be associated with individuals or congregations or schools; and in various other ways to afford an infinite variety of interests which can all obtain representation, by gifts, in the work of the hospital. In this way the funds have been extended, and the income improved. The hospital needs more annual subscriptions, and we strongly recommend any one interested in children to make a point of visiting it, for they are likely, in this way, to experience a good deal of pleasure, and to be attracted permanently to the work.

